# Adhesive systems effect over bond strength of resin-infiltrated and de/remineralized enamel

**DOI:** 10.12688/f1000research.20523.1

**Published:** 2019-10-11

**Authors:** Alessandra Buhler Borges, Amjad Abu Hasna, Amanda Guedes Nogueira Matuda, Stephanie Ribeiro Lopes, Ana Paula Valente Pinho Mafetano, Aline Arantes, Angélica Ferreira Duarte, Daphne Camara Barcellos, Carlos Rocha Gomes Torres, Cesar Rogério Pucci

**Affiliations:** 1 Department of Restorative Dentistry, Operative Dentistry, Institute of Science and Technology, São Paulo State University (UNESP), São José dos Campos, São Paulo, 12245-000, Brazil; 2 Department of Restorative Dentistry, Endodontic Division, Institute of Science and Technology, São Paulo State University (UNESP), São José dos Campos, São Paulo, 12245-000, Brazil

**Keywords:** Enamel, Adhesives, Remineralization, Demineralization, Resin infiltration.

## Abstract

**Background: **The purpose of this study was to evaluate the effect of different bonding agents on bond-strength to demineralized enamel after remineralizing treatments and resin infiltration.

**Methods: **Buccal enamel of 120 bovine incisors was polished and then were divided into five experimental groups: SE (sound enamel); DE (demineralized enamel); AS (demineralized enamel immersed in artificial saliva for eight weeks); NaF (demineralized enamel treated with 0.05% sodium fluoride solution (one minute) for eight weeks); Ic (demineralized enamel infiltrated with a low-viscosity resin (Icon-DGM). These groups were subdivided according to adhesive system used: self-etching adhesive Adper Easy One (3M/ESPE) and etch-and-rinse adhesive Single Bond (3M/ESPE). The composite resin blocks were fabricated using a Teflon matrix. A thermomechanical cycling machine was used to carry out the artificial aging of the specimens and thus were sectioned into sticks. The microtensile tests were performed using a universal testing machine at a cross-head speed of 1 mm/min. Data (in MPa) were subjected to two-way ANOVA and Tukey’s tests (5%).

**Results**: Significant differences were found for both factors tested and interactions (p<0.05). Tukey’s test results of µTBS (mean ± SD) were: etch-and-rinse SE (28.79±3.93); DE (30.41±7.22); AS (29.03±3.33); NaF (29.81±4.06)a; Ic (29.47±5.5);  and self-etching SE (30.37±6.96); DE (14.62±4.47); AS (9.79±2.32); NaF (9.36±2.31); Ic (30.78±8.68).

**Conclusions: **Resin infiltration did not affect the bond strength of demineralized enamel for both adhesive systems tested. For etch-and-rinse adhesive, no differences were observed for the tested groups. For self-etching adhesive, only the resin-infiltrated group showed similar bond strength to sound enamel. Both etch-and-rinse and self-etching adhesive systems can be used in resin-infiltrated enamel, if a composite restoration needs to be further performed. In enamel that has undergone the de/remineralization process, the use of a total-etch adhesive might be preferable for the restorative procedure.

## Introduction

Dental caries is a disease that results from the interaction of microbial factors, diet, host factors, and time. When an imbalance occurs in the de/remineralization process on the tooth surface, demineralization takes over and the initial carious lesions occur ( Fejerskov *et al.*, 2015). The submicroscopic changes of initial enamel demineralization include mineral loss from the body of the lesion, with enlargement of intercrystalline spaces and a reduction in subsurface microhardness, whereas the surface remains comparatively highly mineralized ( Featherstone, 2004; Rocha Gomes Torres *et al.*, 2011). The enamel pores cause the characteristic whitish appearance and promote diffusion routes for acids and dissolved minerals ( Paris *et al.*, 2007b; Torres *et al.*, 2012).

Initial enamel carious lesions can regress or even disappear with remineralization treatment ( Featherstone, 2004; Fejerskov *et al.*, 2015; Rocha Gomes Torres *et al.*, 2011). Non-cavitated caries lesions can be repaired by saliva, if there is control of diet and plaque. Additionally, treatment with fluoride is an alternative noninvasive method used for remineralizing carious processes because fluoride improves the acid resistance of enamel and interferes with bacterial metabolism and enzymatic processes. Thus, less invasive treatments have long been adopted to control the progression of initial enamel carious lesions ( Anusavice, 1997; Anusavice, 1998).

The resin infiltration technique is a micro-invasive alternative treatment to prevent the progression of non-cavitated carious lesions. The purpose of low viscosity light-activated resin infiltration is to seal the carious lesion microporosities in order to create a diffusion barrier within the lesion, preventing acids from penetrating into the lesion without resulting in any material on the enamel surface ( Paris *et al.*, 2007b). A number of studies have shown the effectiveness of this technique ( Meyer-Lueckel *et al.*, 2012; Paris *et al.*, 2006; Paris *et al.*, 2010; Paris *et al.*, 2007). The resin matrix is able to strengthen the enamel structure, increasing surface microhardness and thereby preventing enamel surface breakdown ( Anusavice, 1997; Anusavice, 1998; Paris *et al.*, 2007; Paris *et al.*, 2007b; Torres *et al.*, 2012).

The purpose of this study was to evaluate the micro-tensile bond strength (μTBS) between composite resin and demineralized enamel that has been remineralized by different treatments or resin infiltrated, comparing two adhesive systems: self-etching and etch-and-rinse.

## Methods

In this study, 120 bovine incisors were obtained from a slaughterhouse (Mantiqueira - Sao Jose dos Campos - SP - Brazil). Teeth were sectioned 2 mm from the cement-enamel junction to standardize the specimens. The crowns were embedded in acrylic resin and the enamel of the buccal surfaces was worn using abrasive papers (600 grit, FEPA P, Extec, Enfield, CT, USA) coupled to a circular polishing machine (PA-10, Panambra, São Paulo, Brazil) under water-cooling, to expose a standardized area of 6 x 6 mm.

### Enamel demineralization

All specimens were subjected to demineralization (artificial caries), except the control group (sound enamel), and were separately immersed in a solution composed of 50mM acetate buffer solution containing 1.28 mM Ca(NO _3_) _2·_4H _2_O, 0.74 mM NaH _2_PO _4_·2H _2_O, and 0.03 ppm fluorine at a pH of 5.0 for 16 hours at 37ºC. The total volume of solution used was calculated using 2 mL/mm ^2^ of the enamel area ( Queiroz *et al.*, 2008).

Artificial saliva was prepared according to the formulation of ( Göhring *et al.*, 2004) and consisted of hydrogen carbonate (22.1 mmol/L), potassium (16.1 mmol/L), sodium (14.5 mmol/L), hydrogen phosphate (2.6 mmol/L), boric acid (0.8 mmol/L), calcium (0.7 mmol/L), thiocyanate (0.2 mmol/L), and magnesium (0.2 mmol/L), with a final of pH 7.0.

### Experimental groups

The specimens were divided into four groups (n=30) according to the caries treatment used Table 1.

**Table 1.** The experimental groups.

Groups	Enamel type	Storage
**SE (positive control)**	Sound enamel	100mL of deionized water, changed daily for eight weeks
**DE (negative control)**	Demineralized enamel	100mL of deionized water, changed daily for eight weeks
**AS (artificial saliva)**	Demineralized enamel	100mL of artificial saliva, changed daily for eight weeks
**NaF (0.05% fluoride** **solution)**	Demineralized enamel	Immersed daily for one minute in 1mL of 0.05% NaF solution for eight weeks, then rinsed with deionized water and stored in artificial saliva
**Ic (resin infiltration)**	Demineralized enamel was resin infiltrated (Icon, DMG, Hamburg, Germany)	100mL of artificial saliva, changed daily for eight weeks

In the resin infiltration (Ic) group, the infiltration procedure was carried out in accordance with the manufacturer’s instructions: (1) Icon-Etch (15% hydrochloric acid) was applied for two minutes and then the specimens were rinsed with water and air dried for 30 seconds; (2) Icon-Dry (ethanol) was applied for 30 seconds and air dried; (3) Icon-Infiltrant was applied two times, the first time for three minute and the second time for one minute; (4) both applications were light cured for 40 seconds; (5) specimens were polished with aluminum oxide abrasive papers (4000 grit; FEPA-P, Extec) for 20 seconds, to remove the surplus material.

### Restorative procedures

Two adhesive systems, namely the etch-and-rinse technique, using two layers of Adper Single Bond/SB total-etch adhesive (3M ESPE, St. Paul, MN, USA), and the one-step self-etching technique, using Adper Easy One one-step self-etching adhesive system (3M ESPE), were used in each group (n=15), as used in the study of ( Gupta *et al.*, 2017).

Composite resin blocks (Filtek Z350, 3M ESPE) (4mm high) were built on the treated surfaces of enamel using a Teflon mold. Every 2mm portion was light cured for 40 seconds. The bonded specimens were stored in distilled water (37ºC for 24 h).

The specimens were then subjected to thermo-mechanical wear (37000 ER machine, ERIOS, São Paulo, SP, Brazil). Mechanical cycling was performed with load of 60 N and 100,000 cycles. The load was applied on the composite resin restoration, perpendicular to the enamel surface. Simultaneously, thermal cycling was performed with distilled water at temperatures of 5ºC, 37ºC and 55ºC for 30 seconds at each temperature, totaling 10,000 cycles ( Bedran-de-Castro *et al.*, 2004).

Parallel sections measuring approximately 1mm ^2^ were used, as in the study of ( Farias de Lacerda *et al.*, 2016), and μTBS tests were performed in a universal testing machine (DL-1000, EMIC, São José dos Pinhais, PR, Brazil), with a 10 kg load cell, at a cross-head speed of 0.5 mm/min, in accordance with the ISO 11405 Standard. The μTBS data were expressed in megapascal (MPa).

Qualitative analysis was performed with stereomicroscopy (Discovery V20, Germany) at 20× magnification for failure mode analysis of each specimen. Failure mode was classified as:

(1) Predominant cohesive in composite (composite resin failure)
(2) Predominant cohesive in enamel (enamel failure)
(3) Adhesive in the interface enamel/composite (failure only in the interface)
(4) Mixed (mixed failure between composite resin -enamel and cohesive in composite or cohesive in enamel)

### Statistical analysis

Sticks that presented cohesive failure were discarded. The mean value for the sticks originating from each tooth was calculated and used for the statistical analysis. Data were analyzed by two-way ANOVA (enamel treatment, adhesive system) followed by the Tukey’s test (α = 5%) using GraphPad Prism 6 software (GraphPad Software, San Diego, CA, USA).

### Scanning electron microscopy (SEM) examination

Specimens were sectioned perpendicularly to the bonding interface for SEM analysis. The sections were polished with 2000 and 4000 mesh abrasives. Phosphoric acid etchant was applied for five seconds and rinsed off with water for 10 seconds. Specimens were dehydrated, sputter-coated with gold-palladium and examined by SEM (Inspect S50, FEI, Hillsboro, Oregon, USA) operated at 15 kV ( Farias de Lacerda *et al.*, 2016).

## Results

Table 2 presents the results of the Tukey’s test for the factor ‘ *adhesive system’*. It can be observed that etch-and-rinse showed significantly higher values than self-etching ( Abu Hasna, 2019).

**Table 2.** Tukey’s test (5%) comparison of bond strength means (MPa) and Standard Deviation (±SD) for the factor “ *adhesive system*”.

*Adhesive system*	*Mean (±SD)*	*Homogeneous* *sets*
Self-etching	18.98 (±4.95)	a
Etch-and-rinse	29.50 (±4.81)	b

*Mean values with different letters show significant difference.

Table 3 shows the results of the Tukey’s test for the factor ‘ *enamel treatment’*. Positive control and resin infiltration groups showed significantly higher values than the negative control, 0.05% fluoride solution and artificial saliva groups (p<0.05).

**Table 3.** Tukey’s test (5%) comparison of bond strength means (MPa) and Standard Deviation (±SD) for the factor “ *enamel treatment*”.

Enamel treatment	Mean (±SD)	Homogeneous sets
Artificial Saliva	19.41 (±2.82)	a
0.05% Fluoride Solution	19.58 (±3.18)	a
Negative control	22.52 (±5.84)	a
Positive Control	29.58 (±5.44)	b
Resin Infiltration	30.13 (±7.09)	b

*Mean values with same letters show there was no statistically significant difference.

**Figure 1.**
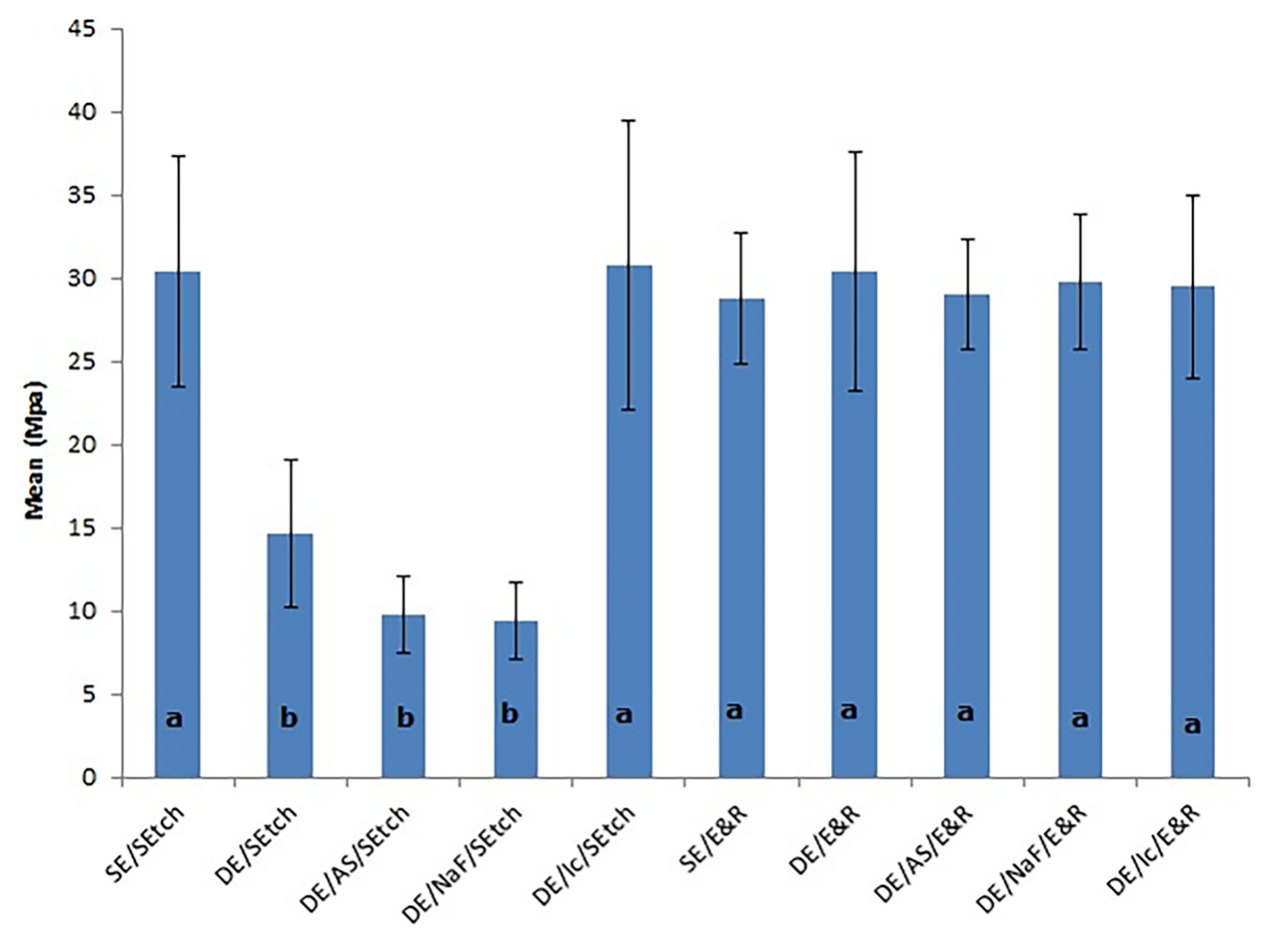
Means (SD) of the μTBS values obtained for both adhesives after different treatments and results. The letters denote significant differences among the groups. SE, sound enamel; DE, demineralized enamel; AS, artificial saliva; NaF, sodium fluoride; Ic, resin infiltration; SEtch, self-etching; E&R, etch-and-rinse.

All groups showed significantly higher μTBS values than self-etching adhesive bonded to demineralized enamel that had been remineralized by saliva or sodium fluoride and bonded to negative control (demineralized enamel that received no remineralizing treatment) ( Figure1).

**Figure 2.**
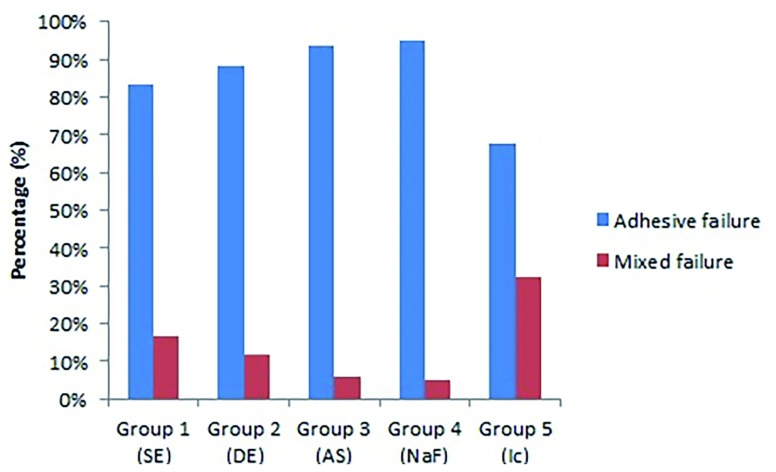
Graphic representation of the number of failures (%) for the groups tested, according to the treatments. SE, sound enamel; DE, demineralized enamel; AS, artificial saliva; NaF, sodium fluoride; Ic, resin infiltration.

The adhesive failures were predominant in all experimental groups ( Figure 2). 

**Figure 3.**
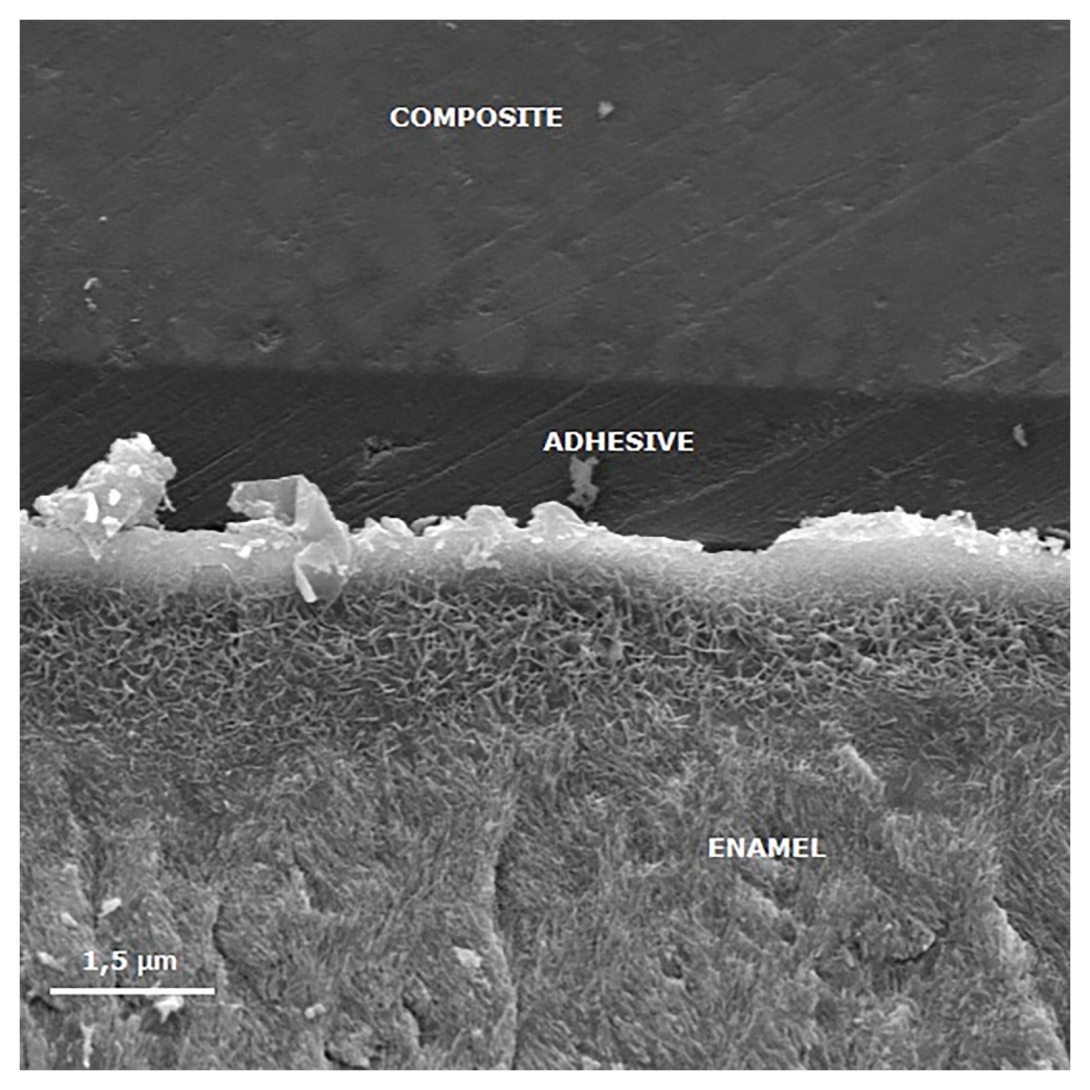
Scanning electron micrograph (2000×) of the bond interface of demineralized enamel (negative control) and Adper Easy Bond. The porous enamel subsurface remains visible because demineralized enamel could not be completely infiltrated by the self-etching adhesive system.

Figures 3 to Figure 6 show SEM images obtained from the interfaces of the self-etching treated.

**Figure 4.**
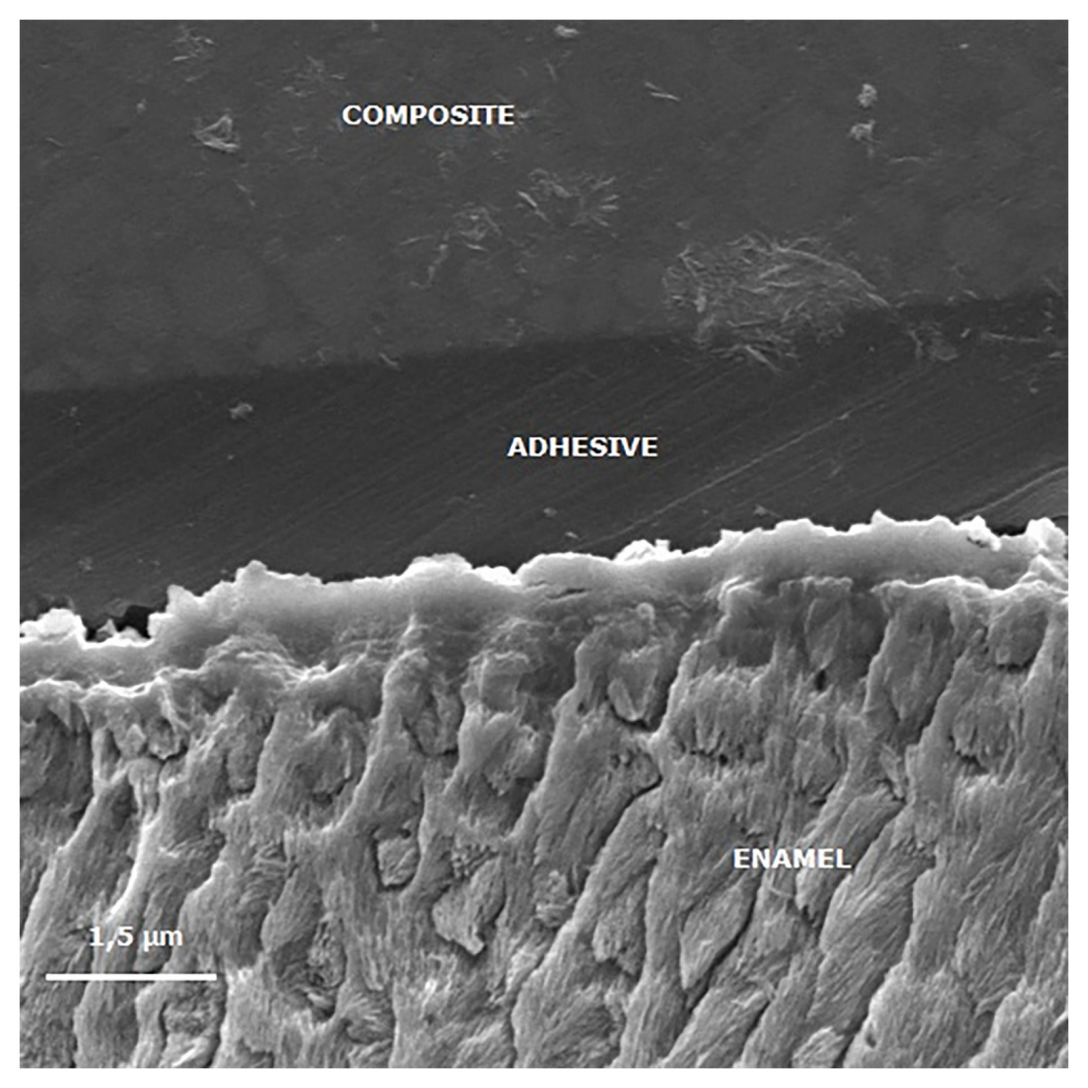
Scanning electron micrograph (2000×) of the bond interface of demineralized enamel that received artificial saliva remineralizing treatment and Adper Easy Bond. The image shows reduced enamel penetration of the self-etching adhesive system.

**Figure 5.**
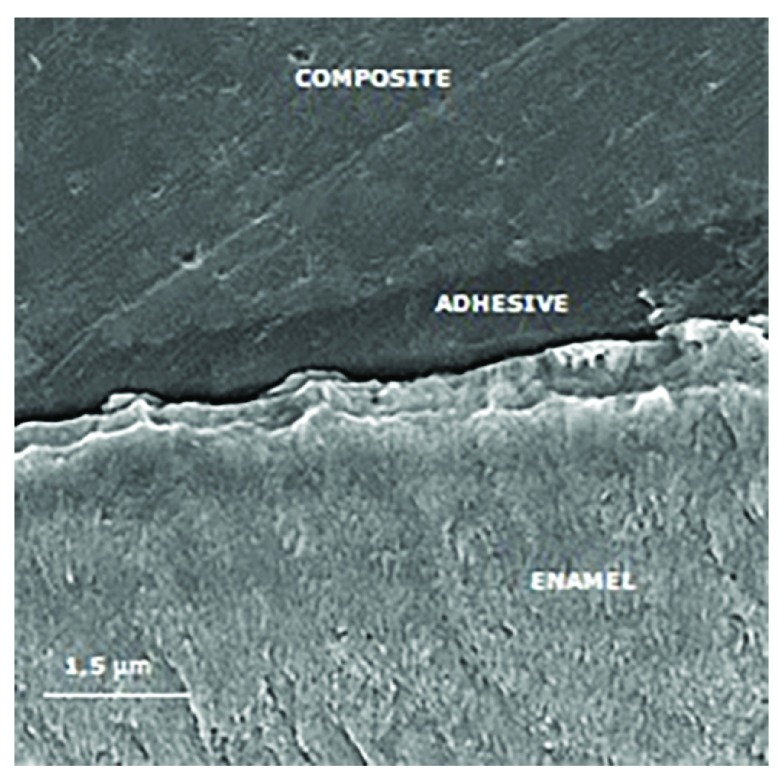
Scanning electron micrograph (2000×) of the bond interface of demineralized enamel that received sodium fluoride remineralizing treatment and Adper Easy Bond. It is observed a poor adhesive penetration, with a fracture line between the adhesive and the treated enamel.

**Figure 6.**
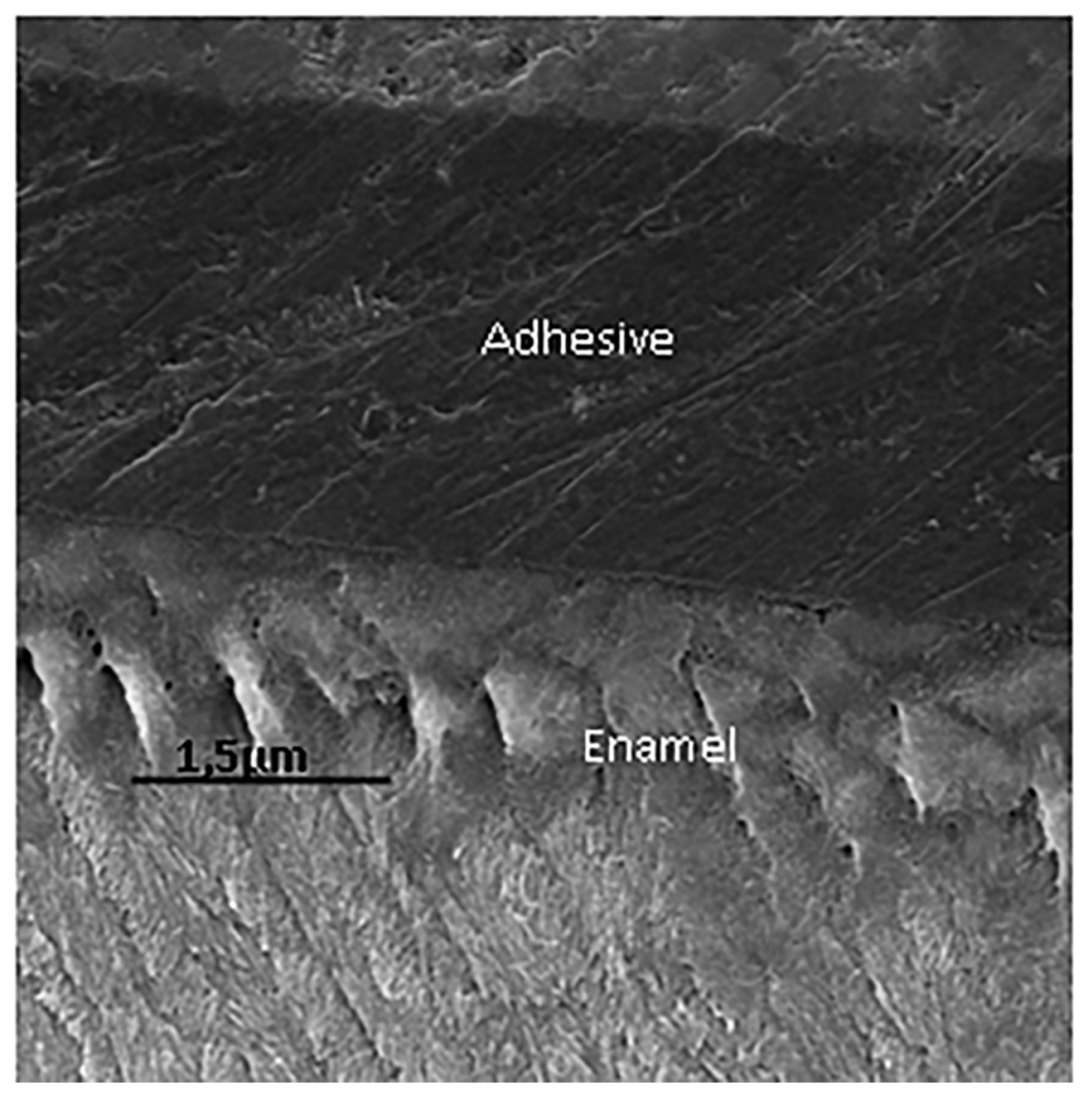
Scanning electron micrograph (2000×) of the bond interface of demineralized enamel after resin infiltration and Adper Easy Bond application. Demineralized enamel was permeated with the infiltrant resin and no distinction between the infiltrant and the adhesive is observed.

## Discussion

This study showed that the etch-and-rinse adhesive system presented superior μTBS means in comparison with the self-etching system. The enamel etch-and-rinse method is based on selective demineralization of the hydroxyapatite crystals founded in tooth enamel, resulting in a highly roughened surface with elevated energy. These features offer better wetting capacity of the resinous monomers that, when polymerized, result in prolongations named tags that ‘anchor’ the resin to the tooth ( Gwinnett, 1981; Torres *et al.*, 2009). In dentistry, the substance most commonly used for this purpose is phosphoric acid at a concentration of 35-37%. Because of its high ionization potential, it results in a final pH of 0.6. As a consequence of the high availability of H+ ions, its application for short periods, such as the 15 seconds usually recommended, is capable of producing a suitable enamel etching pattern ( Barkmeier *et al.*, 1986), resulting in exceptional micromechanical interlocking by the tags created ( Torres *et al.*, 2009).

Acidic adhesive agents, called self-etchants, were introduced with the objective of promoting simultaneous demineralization and impregnation of the substrate. The methacrylated phosphoric ester present in the Adper Easy One adhesive system is used for this purpose. The self-etching adhesive systems were selected depending on their pH values ( Poggio *et al.*, 2014; Van Meerbeek *et al.*, 2003), and Adper Easy One is classified as mild (pH >2.0). Therefore, the concentration of this adhesive solution in an aqueous solution and the number of ionizable radicals are lower in comparison with those in phosphoric acid, and consequently, the etching capacity of this adhesive system is more restricted. As a result, this one-step adhesive does not demineralize enamel to the same extent as phosphoric acid does, promoting a less microretentive surface and usually lower μTBS values ( Poggio *et al.*, 2014).

Moreover, according to ( Erickson *et al.*, 2009), phosphoric acid promotes a particular morphology of the resin-enamel that permits fairly extensive resin penetration, creating a three-dimensional structure (i.e. scalloped), and the transition from resin to sound enamel is distributed over a variety of microns. This interface can be more resistant to crack propagation when compared with the relatively planar interface promoted by self-etch adhesives.

The groups treated with artificial saliva and sodium fluoride showed the lowest μTBS values when associated with self-etching Adper Easy One. Saliva contains calcium (Ca) and phosphate (Pi) in supersaturated concentrations, and these ions are continually deposited or re-deposited on the enamel surface that has suffered loss of these ions ( Cury & Tenuta, 2009). The fluoride ions can also remain incorporated into remineralizing enamel, mainly in surface lesions, changing the carbonated apatite to a fluoroapatite-like form that is more acid tolerant and makes more acid resistant hard tissues ( Cury & Tenuta, 2009; Lee *et al.*, 2010; Rocha Gomes Torres *et al.*, 2011). A previous study showed that both saliva exposure for eight weeks and daily sodium fluoride treatment resulted in increased surface microhardness of demineralized specimens ( Rocha Gomes Torres *et al.*, 2011). It is therefore hypothesized that the increased acid resistance of remineralized enamel impaired the conditioning effect of the moderate self-etching system tested, thus promoting a less microretentive surface, as shown in Figure 2, and consequently, lower μTBS values. Additionally, inactive lesions have thick surface layers compared with active lesions ( Neuhaus *et al.*, 2013). These remineralizing treatments may have promoted thick surface layers of inactive lesions, inhibiting penetration of both the acidic and resinous monomers of Adper Easy One into the lesion.

When the etch-and-rinse system was used, no difference was observed between sound and demineralized enamel bonding, as also shown previously ( Wiegand *et al.*, 2011). Nevertheless, the demineralized enamel presented lower μTBS values than the sound specimens when the self-etching system was applied, in accordance with a previous study ( Jia *et al.*, 2012). Similar to the groups remineralized with artificial saliva and fluoride, this result might be explained by differences in lesion structures, in particular with regard to the surface layer. The surface layer of an initial enamel carious lesion has a higher mineral content in comparison with the underlying body of the lesion ( Cury & Tenuta, 2009; Torres *et al.*, 2012). Therefore, this surface layer can form a barrier, hampering infiltration into the lesion body. In order to increase surface layer porosity, acid etching has been considered to make the underlying body of lesion accessible ( Paris *et al.*, 2007b). The etching capacity of Adper Easy One may not be effective in degrading the surface layer, due to the higher pH and lower acidic capacity of this mild self-etch adhesive, as also reported previously with a self-etching system ( Mueller *et al.*, 2006). This would result in a shallow inter-crystallite infiltration of the adhesive and a lack of inter-prismatic resin tag formation ( Erickson *et al.*, 2009), with remaining non-infiltrated porosities ( Figure 3), which could be a possible explanation for the less effective bonding of Adper Easy One to demineralized enamel.

The groups infiltrated with the low-viscosity resin and bonded with Adper Easy Bond or Single Bond adhesives showed similar μTBS values to the positive control group (sound enamel). The resin infiltrant contains monomers with high penetration coefficients and adequate hardening ( Paris *et al.*, 2013). The satisfactory μTBS values associated with infiltrated groups may have been optimized due to the affinity between the monomers present in the infiltrant and the monomers of the adhesive systems. According to the results of the present study, resin infiltration is compatible with both total-etch and self-etch adhesives, and therefore, restorative treatment can be indicated on tooth surfaces treated with resin infiltration, since it does not negatively interfere in the composite bond to enamel. Previous studies also showed that the application of an etch-and-rinse adhesive after resin infiltration did not alter enamel μTBS ( Wiegand *et al.*, 2011), or even increase the adhesion of a self-etching adhesive ( Jia *et al.*, 2012).

In order to infiltrate a caries lesion, resin infiltration requires the application of 15% hydrochloric acid to promote erosion of the surface layer and allow the resin to penetrate into the porous spaces of the lesion body ( Paris *et al.*, 2006; Torres *et al.*, 2012). An appropriate acid etching pattern enhances resin infiltration into the more porous lesion body structures, both in natural caries lesions ( Paris *et al.*, 2006) and also in artificial lesions ( Belli *et al.*, 2011), optimizing the μTBS to the substrate, as was observed in this study, in which the infiltrated groups reestablished the μTBS to the levels achieved in the sound enamel ( Wiegand *et al.*, 2011).

In order to increase the penetration coefficient of the resin on porous enamel subsurface, a high content of TEG-DMA monomer is added to the infiltrant low-viscosity resin ( Paris *et al.*, 2007a). On the other hand, this monomer has been related to increased susceptibility to degradation of the resin over time ( Munksgaard & Freund, 1990). In this study, thermo-mechanical artificial aging was performed; nevertheless, this did not seem to influence the μTBS of the infiltrated/bonded groups, as also observed in a previous study ( Jia *et al.*, 2012), since these groups exhibited μTBS means comparative to the positive control group.

According to the above, resin infiltration is compatible with both total-etch and self-etch adhesives; thus, restorative treatment can be indicated on tooth surfaces treated with resin infiltration, since it does not negatively interfere with the composite bond to enamel.

The low-viscosity resin infiltration treatment did not affect enamel μTBS values both for the single-step self-etching and the conventional two-step self-etching adhesive systems. The demineralization and remineralization treatments reduced enamel μTBS values of the self-etching adhesive tested.

## Data availability

### Underlying data

Harvard Dataverse: Replication Data for: Adhesive systems effect over bond strength of resin-infiltrated and de/remineralized enamel. https://doi.org/10.7910/DVN/V3WF3M ( Abu Hasna, 2019).

This project contains the following underlying data:

- Raw Data 1.tab (raw bond strength values for all groups with self-etching adhesive system)
- Raw Data 2.tab (raw bond strength values for all groups with etch-and-rinse)
- Figure 3.tiff – Figure 6.tiff (unedited scanning electron microscopy images)

Data are available under the terms of the Creative Commons Zero "No rights reserved" data waiver (CC0 1.0 Public domain dedication).
