# Supplementary material for: Adhesive systems effect over bond strength of resin-infiltrated and de/remineralized enamel
Source: F1000Res. 2019 Oct 11;8:1743. [Version 1] doi: 10.12688/f1000research.20523.1 (PMC6833993; doi:10.12688/f1000research.20523.1)
Supplement: Peer review history

# Peer review: Reviewer response for version 1

**DOI:** 10.5256/f1000research.22557.r55146

**Published:** 2019-11-05

**Authors:** Talal Al-Nahlawi

**Affiliations:** 1 Department of Endodontics and Operative Dentistry, Syrian Private University, Damascus, Syria

The Abstract was well written and fully comprehensive, however this phrase should be corrected:

 ...; NaF (demineralized enamel treated with 0.05% sodium fluoride solution (one minute) for eight weeks);...

 Should mention that this immersion was done DAILY for one minute and lasts 8 weeks.

 The introduction presents the studied issue through a brief literature review. The objective was clear in the bottom of the introduction. However, I suggest for the authors to include the null hypothesis of the this study.  

 The methods:

 As a suggestion, this paragraph (The specimens were then subjected to thermo-mechanical wear (37000 ER machine, ERIOS, São Paulo, SP, Brazil). Mechanical cycling was performed with load of 60 N and 100,000 cycles. The load was applied on the composite resin restoration, perpendicular to the enamel surface.) may be rewritten as the following: (The specimens were then subjected to thermo-mechanical cycling (37000 ER machine, ERIOS, São Paulo, SP, Brazil) with load of 60 N and 100,000 cycles applied on the composite resin restoration, perpendicular to the enamel surface.)

 The discussion, it may be interested to discuss the use of NaF solution instead of using AgF and KF and the immersion for 1 minute instead of for 3 minutes as in the study of Zhi et al ^1^.

 Even more, the discussion of more recent studies could be interested as the study of Alqahtani et al ^2^.

 The study was well written, the objective is interested and has a relevance in this area. The results were promising and the study and the authors collaborated in the understanding of the behavior of many materials in the de/remineralization process on the tooth surface. The conclusions of this paper drawn adequately supported by the results.

I confirm that I have read this submission and believe that I have an appropriate level of expertise to confirm that it is of an acceptable scientific standard.

## References

[1] : An in vitro study of silver and fluoride ions on remineralization of demineralized enamel and dentine. *Aust Dent J*.2013;58(1) : 10.1111/adj.12033 50-6 10.1111/adj.12033 23441792

[2] : Effect of diode laser and topical fluoride applications on white-spot lesions in bovine enamel. *Gen Dent*.67(6) :45-51 31658024

# Peer review: Reviewer response for version 1

**DOI:** 10.5256/f1000research.22557.r55147

**Published:** 2019-11-04

**Authors:** Heleine Maria Chagas Rêgo

**Affiliations:** 1 Schulich School of Medicine & Dentistry, Western University, London, ON, Canada

The article establishes a guide to the protocol that should be used to improve the bond strength of adhesive systems to the de/remineralized enamel surface. The subject is relevant, once that the use of the adhesive technique is a common procedure in the dental office and any change on the dental subtract can affect the effectives and longevity of the restauration.

 Abstract

 The results and conclusion in the abstract are clear and related to the objective of the study.

 Introduction

 The authors should have explained that the choice of treatment for caries lesions is not only related to the progress and location of the injury, but also to the patient's caries risk.

 It would have been interesting if they had addressed the importance of studying the bond strength of the adhesive systems to the enamel surface and the differences (possible vantages and disadvantages) of the adhesive systems that were used in the study, this would provide a stronger support for the relevance of the study and a link with the discussions and possible results.

 Methodology

 Good study design with clear and detailed methodology.

 References should have been cited to support the use of bovine tooth considering its similarity to the human tooth. It would have been relevant to establish the correlation between the number of thermo-mechanical cycles used and the correspondent period of activity in the oral cavity.

 Results

 The results of the Tukey’s test are clear and showed that the etch-and-rinse adhesive system performed better than the self – etch adhesive system, and that the use of the resin infiltration was effective to maintain a good bond strength for both types of adhesive systems used, been equivalent to the control group.

 The authors should have used SEM images of the control groups of the two adhesive systems, as this should be the standard to be achieved, thus clarifying the effectiveness of the resin infiltration in maintaining the bond strength of the treated enamel.

 Discussion

 The discussion is consistent with the results of the study and the authors were able to justify the good performance of the resin infiltration and the limitations regarding the use of the self - etching adhesive system when compared to the etch-and-rinse adhesive system.

 Review the last paragraph of the discussion;

 Instead: '…and the conventional two-step self-etching adhesive systems…'

 I believe that it should be: '…and the conventional two-step total-etching adhesive systems…'

I confirm that I have read this submission and believe that I have an appropriate level of expertise to confirm that it is of an acceptable scientific standard.
